# Analytical Solution to a Third-Order Rational Difference Equation

**DOI:** 10.1155/2023/8971590

**Published:** 2023-04-05

**Authors:** Alvaro H. Salas S., Gilder Cieza Altamirano, Rafaél Artidoro Sandoval Núñez

**Affiliations:** ^1^Universidad Nacional de Colombia, Fizmako Research Group, Bogotá, Colombia; ^2^Universidad Nacional Autónoma de Chota, Cajamarca, Peru; ^3^Fizmako Research Group, Bogotá, Colombia

## Abstract

Inspired by some open conjectures in a rational dynamical system by G. Ladas and Palladino, in this paper, we consider the problem of solving a third-order difference equation. We comment the conjecture by Ladas. A third-order rational difference equation is solved analytically. The solution is compared with the solution to the linearized equation. We show that the solution to the linearized equation is not good, in general. The methods employed here may be used to solve other rational difference equations. The period of the solution is calculated. We illustrate the accuracy of the obtained solutions in concrete examples.

## 1. Introduction

The use of recurrences to solve mathematical problems dates back to Babylon in 2000 B.C.E. in the context of the approximate resolution of algebraic equations and the approximate calculation of square roots. In Greek times, the Pythagoreans (fifth century B.C.E.) implicitly used nonautonomous difference equations to study the numbers associated with figures or pentagonal numbers.

The Fibonacci sequence, continued fractions, binomial coefficients, the calculus of finite differences, the Newton–Raphson method, and the numerical methods to approximate the solutions of a differential equation are just some of them (see [1, 2] for more details). In the first half of the twentieth century, great interest arose in the development of numerical methods, which was greatly enhanced by the appearance of powerful computer calculation tools.

In the 50s of the last century, moreover, nonlinear difference equations began to be used as applied models, especially in ecology. Later, the discovery that even the simplest models exhibit enormous complexity led to the introduction of mathematical chaos and renewed interest in the theory of difference equations.

Nonlinear difference equations and their systems are hot topics that have attracted the attention of several researchers. A significant number of papers are devoted to this field of research. One can consult, for example, the papers[3–10], where one can find concrete models of such equations and systems, as well as understand the techniques used to solve them and investigate the behavior of their solutions.

Recently, an increased interest has been witnessed in studying the theory of discrete dynamical systems, specifically of their associated difference equations. A sizable number of works on the behavior and properties of pertaining solutions (boundedness and unboundedness) have been published in various areas of applied mathematics and physics. The theory of difference equations finds many applications in almost all areas of natural science [11]. The difference equations with discrete and continuous arguments play important role for understanding nonlinear dynamics and phenomena [8]. The increased interest in difference equations is partly due to their ease of handling.

Although difference equations have very simple forms, it is extremely difficult to completely understand the global behavior of their solutions. One can refer to [4–6] and the references therein. Difference equations have always played an important role in the construction and analysis of mathematical models of biology, ecology, physics, and economic processes. The study of nonlinear rational difference equations of higher order is of paramount importance, since we still know little about such equations.

Let *m* ≥ 1 be a natural number. Given *f* : *R*^*m*^⟶*R* which we will call iteration function, a difference equation (DE) of order *m* in explicit form is any expression like the following:

(1) $$\begin{array}{c} x_{n+1}=f\left(x_{n-m+1},\ldots ,x_{n}\right)\text{.} \end{array}$$

The above formula allows us to build a family of sequences called the set of solutions of the DE, whose definition is as follows: fixed a vector *X*=(*x*_−*m*+1_,…, *x*_0_), the solution of (1) from initial conditions *X* or generated by the initial conditions *X* is the sequence (*x*_*j*_)_*j*=−*m*+1_^*∞*^ whose first *m* terms are the components of *X* and the rest are obtained inductively by formula (1). When for some *r* ≥ 0, the vector (*x*_*r*−*m*+1_,…, *x*_*r*_) does not belong to the domain of definition of *f*, the construction of (*x*_*j*_)_*j*=−*m*+1_^*∞*^ cannot be realized. In such a case, we say that *X* is an element of the forbidden set of (1), denoting it by *𝒫*.

The expression solution of the difference equation is reserved for the sequences generated from the elements of *ℬ*=*R*^*n*^/*𝒫*, called the good set of the DE. Occasionally, the term finite solution is used. When *X* ∈ *𝒫* and *r* is the largest integer such that *x*_*m*_ is well defined, refer to (*x*_*j*_)_*j*=−*m*+1_^*r*^. But, unless otherwise indicated, the word “solution” is associated with sequences of infinite terms. To emphasize this difference, we will sometimes say that such solutions are well defined. Solutions of a DE are also called trajectories or orbits. Such nominations are inspired by the terminology of dynamic systems.

In this paper, we will consider the following third-order rational difference equation:

(2) $$\begin{array}{c} x_{n+1}=\frac{a_{0}+a_{1}x_{n}+a_{2}x_{n-1}+a_{3}x_{n-2}}{b_{0}+b_{1}x_{n}+b_{2}x_{n-1}+b_{3}x_{n-2}}\text{,} \end{array}$$

(3) $$\begin{array}{c} X=\left(x_{-2},x_{-1},x_{0}\right)=\left(c_{-2},c_{-1},c_{0}\right)\text{.} \end{array}$$

The most important solutions to equation (2) are the periodic solutions, those formed by a quantity finite number of terms which repeat itself indefinitely. Their relevance lies in the fact that, on many occasions, the equation can be described qualitatively by identifying its periods and the behavior of the rest of the solutions with respect to them. For example, a common situation is that some periods behave as attractors of the rest of the solutions, which implies that the model associated with DE will consist, in the long run, of a certain cycle.

Even when the dynamics of the ED are not so clear, the determination of the periodic solutions is still relevant information to give us an idea of what is happening.

In [7],Abo-Zeid has discussed the global behavior of all solutions of the difference equation:

(4) $$\begin{array}{c} x_{n+1}=\frac{x_{n}x_{n-1}}{ax_{n}+bx_{n-1}}\text{,} \end{array}$$

where *a* and *b* are real numbers and the initial conditions *x*_1_ and *x*_0_ are real numbers. A class of third-order rational difference equations of form (2) with nonnegative coefficients is considered in [12].

## 2. The Solution

We seek approximate analytical solution in the ansatz form

(5) $$\begin{array}{c} x_{n}=\mu_{0}+\mu_{1}r_{1}^{n}+\mu_{2}r_{2}^{n}+\mu_{3}r_{3}^{n}\\ +\mu_{1,1,0}r_{1}^{n}r_{2}^{n}+\mu_{1,0,1}r_{1}^{n}r_{3}^{n}+\mu_{0,1,1}r_{2}^{n}r_{3}^{n}\\ +\mu_{2,0,0}r_{1}^{2n}+\mu_{0,2,0}r_{2}^{2n}+\mu_{0,0,2}r_{3}^{2n}\text{.} \end{array}$$

We define the residual as

(6) $$\begin{array}{c} R\left(n\right)=\left(b_{0}+b_{1}x_{n+2}+b_{2}x_{n+1}+b_{3}x_{n}\right)x_{n+3}+\left(a_{0}+a_{1}x_{n+2}+a_{2}x_{n+1}+a_{3}x_{n}\right)\text{.} \end{array}$$

Then,

(7) $$\begin{array}{c} R\left(n\right)=\kappa_{0}+P\left(r_{1}\right)z_{1}+P\left(r_{2}\right)z_{2}+P\left(r_{3}\right)z_{3}\\ +\kappa_{1,1,0}z_{1}z_{2}+\mu_{1,0,1}z_{1}z_{3}+\mu_{0,1,1}z_{2}z_{3}\\ +\kappa_{2,0,0}z_{1}^{2}+\kappa_{0,2,0}z_{2}^{2}+\kappa_{0,0,2}z_{3}^{2}+\cdots \text{,} \end{array}$$

where *z*_*j*_=*r*_*j*_^*n*^(*j*=1,2,3) and

(8) $$\begin{array}{c} \kappa_{0}=a_{0}+\left(a_{1}+a_{2}+a_{3}+b_{0}\right)\mu_{0}+\left(b_{1}+b_{2}+b_{3}\right)\mu_{0}^{2}\text{,}\\ P\left(\zeta \right)=a_{3}+b_{3}\mu_{0}+\left(a_{2}+b_{2}\mu_{0}\right)\zeta +\left(a_{1}+b_{1}\mu_{0}\right)\zeta^{2}+\left(b_{0}+\left(b_{1}+b_{2}+b_{3}\right)\mu_{0}\right)\zeta^{3}\text{,}\\ \kappa_{2,0,0}=a_{3}\mu_{2,0,0}+a_{1}r_{1}^{4}\mu_{2,0,0}+a_{2}r_{1}^{2}\mu_{2,0,0}+b_{3}\mu_{0}\mu_{2,0,0}+b_{0}r_{1}^{6}\mu_{2,0,0}+b_{1}\mu_{0}r_{1}^{6}\mu_{2,0,0}\\ +b_{2}\mu_{0}r_{1}^{6}\mu_{2,0,0}+b_{3}\mu_{0}r_{1}^{6}\mu_{2,0,0}+b_{1}\mu_{0}r_{1}^{4}\mu_{2,0,0}+b_{2}\mu_{0}r_{1}^{2}\mu_{2,0,0}+b_{1}\mu_{1}^{2}r_{1}^{5}+b_{2}\mu_{1}^{2}r_{1}^{4}+b_{3}\mu_{1}^{2}r_{1}^{3},\\ \kappa_{0,2,0}=a_{3}\mu_{0,2,0}+a_{1}r_{2}^{4}\mu_{0,2,0}+a_{2}r_{2}^{2}\mu_{0,2,0}+b_{3}\mu_{0}\mu_{0,2,0}+b_{0}r_{2}^{6}\mu_{0,2,0}+b_{1}\mu_{0}r_{2}^{6}\mu_{0,2,0}\\ +b_{2}\mu_{0}r_{2}^{6}\mu_{0,2,0}+b_{3}\mu_{0}r_{2}^{6}\mu_{0,2,0}+b_{1}\mu_{0}r_{2}^{4}\mu_{0,2,0}+b_{2}\mu_{0}r_{2}^{2}\mu_{0,2,0}+b_{1}\mu_{2}^{2}r_{2}^{5}+b_{2}\mu_{2}^{2}r_{2}^{4}+b_{3}\mu_{2}^{2}r_{2}^{3},\\ \kappa_{0,0,2}=a_{3}\mu_{0,0,2}+a_{1}r_{3}^{4}\mu_{0,0,2}+a_{2}r_{3}^{2}\mu_{0,0,2}+b_{3}\mu_{0}\mu_{0,0,2}+b_{0}r_{3}^{6}\mu_{0,0,2}+b_{1}\mu_{0}r_{3}^{6}\mu_{0,0,2}\\ +b_{2}\mu_{0}r_{3}^{6}\mu_{0,0,2}+b_{3}\mu_{0}r_{3}^{6}\mu_{0,0,2}+b_{1}\mu_{0}r_{3}^{4}\mu_{0,0,2}+b_{2}\mu_{0}r_{3}^{2}\mu_{0,0,2}+b_{1}\mu_{3}^{2}r_{3}^{5}+b_{2}\mu_{3}^{2}r_{3}^{4}+b_{3}\mu_{3}^{2}r_{3}^{3},\\ \kappa_{1,1,0}=a_{3}\mu_{1,1,0}+a_{1}r_{2}^{2}r_{1}^{2}\mu_{1,1,0}+a_{2}r_{2}r_{1}\mu_{1,1,0}+b_{3}\mu_{0}\mu_{1,1,0}\\ +b_{0}r_{2}^{3}r_{1}^{3}\mu_{1,1,0}+b_{1}\mu_{0}r_{2}^{3}r_{1}^{3}\mu_{1,1,0}+b_{2}\mu_{0}r_{2}^{3}r_{1}^{3}\mu_{1,1,0}+b_{3}\mu_{0}r_{2}^{3}r_{1}^{3}\mu_{1,1,0}\\ +b_{1}\mu_{0}r_{2}^{2}r_{1}^{2}\mu_{1,1,0}+b_{2}\mu_{0}r_{2}r_{1}\mu_{1,1,0}+b_{1}\mu_{1}\mu_{2}r_{2}^{2}r_{1}^{3}+b_{3}\mu_{1}\mu_{2}r_{1}^{3}\\ +b_{2}\mu_{1}\mu_{2}r_{2}r_{1}^{3}+b_{1}\mu_{1}\mu_{2}r_{2}^{3}r_{1}^{2}+b_{2}\mu_{1}\mu_{2}r_{2}^{3}r_{1}+b_{3}\mu_{1}\mu_{2}r_{2}^{3},\\ \kappa_{1,0,1}=a_{3}\mu_{1,0,1}+a_{1}r_{3}^{2}r_{1}^{2}\mu_{1,0,1}+a_{2}r_{3}r_{1}\mu_{1,0,1}+b_{3}\mu_{0}\mu_{1,0,1}\\ +b_{0}r_{3}^{3}r_{1}^{3}\mu_{1,0,1}+b_{1}\mu_{0}r_{3}^{3}r_{1}^{3}\mu_{1,0,1}+b_{2}\mu_{0}r_{3}^{3}r_{1}^{3}\mu_{1,0,1}+b_{3}\mu_{0}r_{3}^{3}r_{1}^{3}\mu_{1,0,1}\\ +b_{1}\mu_{0}r_{3}^{2}r_{1}^{2}\mu_{1,0,1}+b_{2}\mu_{0}r_{3}r_{1}\mu_{1,0,1}+b_{1}\mu_{1}\mu_{3}r_{3}^{2}r_{1}^{3}+b_{3}\mu_{1}\mu_{3}r_{1}^{3}\\ +b_{2}\mu_{1}\mu_{3}r_{3}r_{1}^{3}+b_{1}\mu_{1}\mu_{3}r_{3}^{3}r_{1}^{2}+b_{2}\mu_{1}\mu_{3}r_{3}^{3}r_{1}+b_{3}\mu_{1}\mu_{3}r_{3}^{3},\\ \kappa_{0,1,1}=a_{3}\mu_{0,1,1}+a_{1}r_{3}^{2}r_{2}^{2}\mu_{0,1,1}+a_{2}r_{3}r_{2}\mu_{0,1,1}+b_{3}\mu_{0}\mu_{0,1,1}\\ +b_{0}r_{3}^{3}r_{2}^{3}\mu_{0,1,1}+b_{1}\mu_{0}r_{3}^{3}r_{2}^{3}\mu_{0,1,1}+b_{2}\mu_{0}r_{3}^{3}r_{2}^{3}\mu_{0,1,1}+b_{3}\mu_{0}r_{3}^{3}r_{2}^{3}\mu_{0,1,1}\\ +b_{1}\mu_{0}r_{3}^{2}r_{2}^{2}\mu_{0,1,1}+b_{2}\mu_{0}r_{3}r_{2}\mu_{0,1,1}+b_{1}\mu_{2}\mu_{3}r_{3}^{2}r_{2}^{3}+b_{3}\mu_{2}\mu_{3}r_{2}^{3}\\ +b_{2}\mu_{2}\mu_{3}r_{3}r_{2}^{3}+b_{1}\mu_{2}\mu_{3}r_{3}^{3}r_{2}^{2}+b_{2}\mu_{2}\mu_{3}r_{3}^{3}r_{2}+b_{3}\mu_{2}\mu_{3}r_{3}^{3}. \end{array}$$

The number *μ*_0_ is an equilibrium point, and it satisfies the quadratic equation

(9) $$\begin{array}{c} a_{0}+\left(a_{1}+a_{2}+a_{3}+b_{0}\right)\mu_{0}+\left(b_{1}+b_{2}+b_{3}\right)\mu_{0}^{2}=0\text{.} \end{array}$$

The numbers *r*_1_, *r*_2_, and *r*_3_ bare the roots to the cubic equation

(10) $$\begin{array}{c} a_{3}+b_{3}\mu_{0}+\left(a_{2}+b_{2}\mu_{0}\right)\zeta +\left(a_{1}+b_{1}\mu_{0}\right)\zeta^{2}+\left(b_{0}+\left(b_{1}+b_{2}+b_{3}\right)\mu_{0}\right)\zeta^{3}=0\text{.} \end{array}$$

The constants *μ*_*i*,*j*,*k*_ are obtained from the system *κ*_*i*,*j*,*k*_=0(*i*, *j*, *k*=0,1,2). They read

(11) $$\begin{array}{c} \mu_{0,0,2}=-\frac{\mu_{3}^{2}r_{3}^{3}\left(b_{1}r_{3}^{2}+b_{2}r_{3}+b_{3}\right)}{a_{1}r_{3}^{4}+a_{2}r_{3}^{2}+a_{3}+b_{3}\mu_{0}+b_{1}\mu_{0}r_{3}^{6}+b_{2}\mu_{0}r_{3}^{6}+b_{3}\mu_{0}r_{3}^{6}+b_{1}\mu_{0}r_{3}^{4}+b_{2}\mu_{0}r_{3}^{2}+b_{0}r_{3}^{6}}\text{,}\\ \mu_{0,1,1}=-\frac{\mu_{2}\mu_{3}\left(b_{1}r_{3}^{2}r_{2}^{3}+b_{3}r_{2}^{3}+b_{2}r_{3}r_{2}^{3}+b_{1}r_{3}^{3}r_{2}^{2}+b_{2}r_{3}^{3}r_{2}+b_{3}r_{3}^{3}\right)}{a_{1}r_{2}^{2}r_{3}^{2}+a_{2}r_{2}r_{3}+a_{3}+b_{3}\mu_{0}+b_{1}\mu_{0}r_{2}^{3}r_{3}^{3}+b_{2}\mu_{0}r_{2}^{3}r_{3}^{3}+b_{3}\mu_{0}r_{2}^{3}r_{3}^{3}+b_{1}\mu_{0}r_{2}^{2}r_{3}^{2}+b_{2}\mu_{0}r_{2}r_{3}+b_{0}r_{2}^{3}r_{3}^{3}}\text{,}\\ \mu_{0,2,0}=-\frac{\mu_{2}^{2}r_{2}^{3}\left(b_{1}r_{2}^{2}+b_{2}r_{2}+b_{3}\right)}{a_{1}r_{2}^{4}+a_{2}r_{2}^{2}+a_{3}+b_{3}\mu_{0}+b_{1}\mu_{0}r_{2}^{6}+b_{2}\mu_{0}r_{2}^{6}+b_{3}\mu_{0}r_{2}^{6}+b_{1}\mu_{0}r_{2}^{4}+b_{2}\mu_{0}r_{2}^{2}+b_{0}r_{2}^{6}}\text{,}\\ \mu_{1,0,1}=-\frac{\mu_{1}\mu_{3}\left(b_{1}r_{3}^{2}r_{1}^{3}+b_{3}r_{1}^{3}+b_{2}r_{3}r_{1}^{3}+b_{1}r_{3}^{3}r_{1}^{2}+b_{2}r_{3}^{3}r_{1}+b_{3}r_{3}^{3}\right)}{a_{1}r_{1}^{2}r_{3}^{2}+a_{2}r_{1}r_{3}+a_{3}+b_{3}\mu_{0}+b_{1}\mu_{0}r_{1}^{3}r_{3}^{3}+b_{2}\mu_{0}r_{1}^{3}r_{3}^{3}+b_{3}\mu_{0}r_{1}^{3}r_{3}^{3}+b_{1}\mu_{0}r_{1}^{2}r_{3}^{2}+b_{2}\mu_{0}r_{1}r_{3}+b_{0}r_{1}^{3}r_{3}^{3}}\text{,}\\ \mu_{1,1,0}=-\frac{\mu_{1}\mu_{2}\left(b_{1}r_{2}^{2}r_{1}^{3}+b_{3}r_{1}^{3}+b_{2}r_{2}r_{1}^{3}+b_{1}r_{2}^{3}r_{1}^{2}+b_{2}r_{2}^{3}r_{1}+b_{3}r_{2}^{3}\right)}{a_{1}r_{1}^{2}r_{2}^{2}+a_{2}r_{1}r_{2}+a_{3}+b_{3}\mu_{0}+b_{1}\mu_{0}r_{1}^{3}r_{2}^{3}+b_{2}\mu_{0}r_{1}^{3}r_{2}^{3}+b_{3}\mu_{0}r_{1}^{3}r_{2}^{3}+b_{1}\mu_{0}r_{1}^{2}r_{2}^{2}+b_{2}\mu_{0}r_{1}r_{2}+b_{0}r_{1}^{3}r_{2}^{3}}\text{,}\\ \mu_{2,0,0}=-\frac{\mu_{1}^{2}r_{1}^{3}\left(b_{1}r_{1}^{2}+b_{2}r_{1}+b_{3}\right)}{a_{1}r_{1}^{4}+a_{2}r_{1}^{2}+a_{3}+b_{3}\mu_{0}+b_{1}\mu_{0}r_{1}^{6}+b_{2}\mu_{0}r_{1}^{6}+b_{3}\mu_{0}r_{1}^{6}+b_{1}\mu_{0}r_{1}^{4}+b_{2}\mu_{0}r_{1}^{2}+b_{0}r_{1}^{6}}\text{.} \end{array}$$

Finally, the constants *μ*_1_, *μ*_2_, and *μ*_3_ are obtained from the initial conditions

(12) $$\begin{array}{c} x_{-2}=c_{-2}\text{,}\\ x_{-1}=c_{-1}\text{,}\\ x_{0}=c_{0}\text{.} \end{array}$$

## 3. Some Particular Cases

**Example 1 .**

(Ladas–Palladino conjecture). Let us consider the DE

(13) $$\begin{array}{c} x_{n+1}=\frac{\alpha +\beta x_{n}+\gamma x_{n-1}}{x_{n-2}}\;for\;\alpha ,\beta ,\gamma \geq 0\;and\;positive\;initial\;conditions\;x_{-2},x_{-1}\;and\;x_{0}\text{.} \end{array}$$

Ladas–Palladino conjecture claims that the solutions to third-order DE are bounded iff *β*=*γ*. In this case,

(14) $$\begin{array}{c} a_{0}=\alpha \text{,}\\ a_{1}=\beta \text{,}\\ a_{2}=\gamma \text{,}\\ a_{3}=0\text{,}\\ b_{0}=b_{1}=b_{2}=0\text{.}\\ b_{3}=1\text{.} \end{array}$$

Let us find an approximate solution for this DE.

(15) $$\begin{array}{c} \mu_{0}=\frac{1}{2}\left(\sqrt{4\alpha +{\left(\beta +\gamma \right)}^{2}}+\beta +\gamma \right)\text{.} \end{array}$$

The numbers *r*_1_, *r*_2_, and *r*_3_ are the roots to the cubic

(16) $$\begin{array}{c} \mu_{0}\zeta^{3}+\beta \zeta^{2}+\gamma \zeta +\mu_{0}=0\text{.} \end{array}$$

The discriminant to the cubic in (16) equals

(17) $$\begin{array}{c} ∆=\beta^{2}\gamma^{2}-4\mu_{0}\left(\beta^{3}+\gamma^{3}\right)+18\beta \gamma \mu_{0}^{2}-27\mu_{0}^{4}<0\;for\;any\;\alpha ,\beta ,and\;\gamma >0\text{.} \end{array}$$

Thus, we have one real root and two complex roots.

*First case* (*β* ≠ *γ*). In this case, at least one of the roots of the cubic in (16) will have magnitude greater than the unity. The approximate solution will be unbounded. We will not consider this case.
*Second case* (*β*=*γ*). In this case, one of the roots of the cubic in (16) equals −1 and the other two are complex and they lie on the unit circle |*z*|=1. That is, all roots have magnitude 1. The approximate solution will be bounded. In order to simplify the matters, let *x*_*n*_=*z*_*n*_/*β* and *c*=*p*/*β*^2^. Then, the dynamics of (13) can be rewritten as
  (18) $$\begin{array}{c} x_{n+1}=\frac{p+x_{n}+x_{n-1}}{x_{n-2}}, \end{array}$$
just with one parameter *p* ≥ 0. The numbers *r*_1_, *r*_2_, and *r*_3_ are given by
  (19) $$\begin{array}{c} r_{1}=-1\text{,}\\ r_{2}=\cos\;\theta +i\;\sin\;\theta \text{,}\\ r_{3}=\cos\;\theta -i\;\sin\;\theta =r_{2}^{-1}\text{,} \end{array}$$
where
  (20) $$\begin{array}{c} \theta =\tan^{-1}\left(\sqrt{\frac{3p+4\sqrt{p+1}+3}{p+4\sqrt{p+1}+5}}\right)\text{.} \end{array}$$

The solution is written as

(21) $$\begin{array}{c} x_{n}=\mu_{0}+\mu_{1}{\left(-1\right)}^{n}+\mu_{3}R^{-n}+\mu_{2}R^{n}\\ +\frac{\mu_{1}\mu_{2}\left(R^{2}+R+1\right)}{\mu_{0}\left(R^{2}+R+1\right)+R}{\left(-1\right)}^{n}R^{n}+\frac{\mu_{1}\mu_{3}\left(R^{2}+R+1\right)}{\mu_{0}\left(R^{2}+R+1\right)+R}{\left(-1\right)}^{n}R^{-n}\\ -\frac{\mu_{3}^{2}}{\mu_{0}\left(R^{6}+1\right)-R^{2}\left(R^{2}+1\right)}R^{3-2n}-\frac{\mu_{2}^{2}}{\mu_{0}\left(R^{6}+1\right)-R^{2}\left(R^{2}+1\right)}R^{2n+3}\\ +\frac{\mu_{2}\mu_{3}\left(R^{6}+1\right)}{2R^{3}\left(1-\mu_{0}\right)}-\frac{\mu_{1}^{2}}{2-2\mu_{0}}\text{,} \end{array}$$

where

(22) $$\begin{array}{c} R=\cos\;\theta +i\;\sin\;\theta =r_{2}\text{.} \end{array}$$

**Example 2 .**

**Figure 1:**
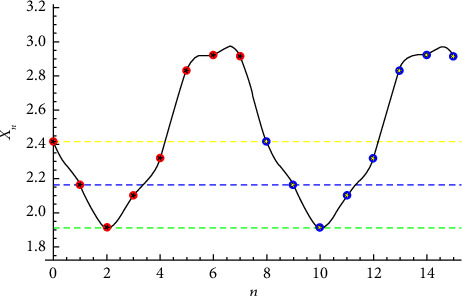
Solution to *x*_*n*+1_=(1+*x*_*n*_+*x*_*n*−1_/*x*_*n*−2_) given that $x_{-2}=1+\sqrt{2},x_{-1}=\left(3/4\right)+\sqrt{2}$, and $x_{0}=\left(1/2\right)+\sqrt{2}$.

**Figure 2:**
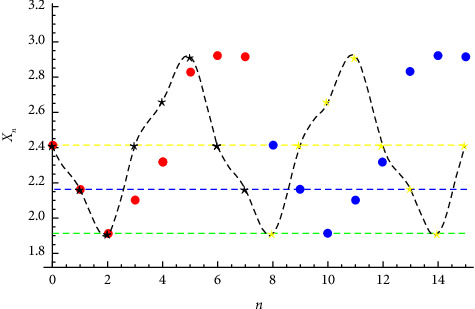
Linearized Solution to *x*_*n*+1_=(1+*x*_*n*_+*x*_*n*−1_/*x*_*n*−2_) given that $x_{-2}=1+\sqrt{2},x_{-1}=\left(3/4\right)+\sqrt{2}$, and $x_{0}=\left(1/2\right)+\sqrt{2}$.

**Figure 3:**
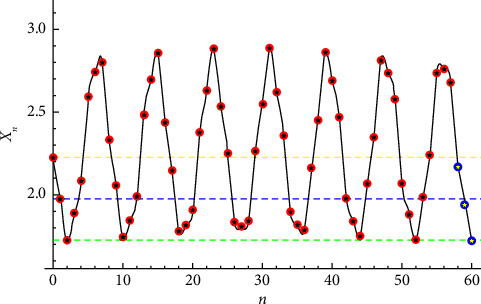
Solution to *x*_*n*+1_=(0.5+*x*_*n*_+*x*_*n*−1_/*x*_*n*−2_) given that *x*_−2_=2.22474, *x*_−1_=1.97474, and *x*_0_=1.72474.

**Figure 4:**
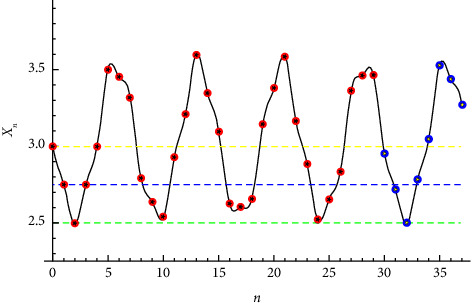
Solution to a third-order difference equation.

Let *p* = 1. This is known as the Todd equation. Let us consider the initial conditions (see Figures1–4)

(23) $$\begin{array}{c} x_{-2}=1+\sqrt{2}\text{,}\\ x_{-1}=\frac{3}{4}+\sqrt{2}\text{,}\\ x_{0}=\frac{1}{2}+\sqrt{2}\text{.} \end{array}$$

The linear approximation $x_{n}=\left(1/12\right)\left({\left(-1\right)}^{n+1}-3\sqrt{3}\sin\left(\pi n/3\right)+\cos\left(\pi n/3\right)+12\sqrt{2}+12\right)$ for this problem is not good (red and blue points correspond to that of Todd's equation solutions).

The approximate solution is given by

(24) $$\begin{array}{c} x_{n}=\left(0.0501063-0.00100115{\left(-1\right)}^{n}\right)\cos\left(\frac{\pi n}{4}\right)-0.0565135{\left(-1\right)}^{n}+0.0103055{\left(-1\right)}^{n}\sin\left(\frac{\pi n}{4}\right)\\ -0.515776\;\sin\left(\frac{\pi n}{4}\right)-0.00535239\;\sin\left(\frac{\pi n}{2}\right)-0.0272878\;\cos\left(\frac{\pi n}{2}\right)+2.44891\text{.} \end{array}$$

The solution is periodic with period *T*=7.

**Example 3 .**

Now, let *p*=0.5 for the initial conditions *x*_−2_=2.22474, *x*_−1_=1.97474, and *x*_0_=1.72474. The solution reads

(25) $$\begin{array}{c} x_{n}=2.26354-0.0140538\;\sin\left(2.2804-1.52026n\right)+0.00814246\;\cos\left(2.2804-1.52026n\right)\\ +\left(0.0489589-0.00101907{\left(-1\right)}^{n}\right)\cos\left(0.760132n\right)-0.0548054{\left(-1\right)}^{n}\\ +\left(0.0110165{\left(-1\right)}^{n}-0.529262\right)\sin\left(0.760132n\right)-0.0101714\;\sin\left(1.52026n+2.2804\right)\\ +0.012663\;\cos\left(1.52026n+2.2804\right)\text{.} \end{array}$$

The solution is periodic with period *T*=57.

Let *p*=3 for the initial conditions *x*_−2_=3, *x*_−1_=2.75, and *x*_0_=2.5. The solution reads

(26) $$\begin{array}{c} x_{n}=3.0257-0.00729324\;\sin\left(2.52321-1.68214n\right)+0.00650347\;\cos\left(2.52321-1.68214n\right)\\ +\left(0.0551657-0.000978734{\left(-1\right)}^{n}\right)\cos\left(0.841069n\right)-0.0608287{\left(-1\right)}^{n}\\ +\left(0.0086946{\left(-1\right)}^{n}-0.490065\right)\sin\left(0.841069n\right)-0.00375297\;\sin\left(1.68214n+2.52321\right)\\ +0.00902229\;\cos\left(1.68214n+2.52321\right)\text{.} \end{array}$$

The solution is periodic with period *T*=29.

## 4. Conclusions and Future Work

We have shown in our paper that approximate analytical solutions of a rational dynamical system, namely, third-order difference equation, are periodic and bounded but this may not happen to the exact solution of such a rational dynamical system. We may use the same methods of linearization to predict orbits and boundedness solutions for other rational dynamical systems such as difference equations of a fourth degree or more, rather than that we may prove or disprove other open conjectures in rational dynamical systems which are proposed by G. Ladas.

## Data Availability

No data were used for supporting this paper.
